# Good social skills despite poor theory of mind: exploring compensation in autism spectrum disorder

**DOI:** 10.1111/jcpp.12886

**Published:** 2018-03-26

**Authors:** Lucy Anne Livingston, Emma Colvert, the Social Relationships Study Team, Patrick Bolton, Francesca Happé

**Affiliations:** ^1^ Social, Genetic and Developmental Psychiatry Centre Institute of Psychiatry, Psychology and Neuroscience King's College London London UK; ^2^ Department of Child and Adolescent Psychiatry Institute of Psychiatry, Psychology and Neuroscience King's College London London UK

**Keywords:** Autism spectrum disorder, compensation, compensatory mechanisms, adaptation, remediation, theory of mind, executive function, camouflaging

## Abstract

**Background:**

It is proposed that some individuals with Autism Spectrum Disorder (ASD) can ‘compensate’ for their underlying difficulties (e.g. in theory of mind; ToM), thus demonstrating relatively few behavioural symptoms, despite continued core cognitive deficits. The mechanisms underpinning compensation are largely unexplored, as is its potential impact on mental health. This study aimed to estimate compensation patterns in ASD, by contrasting overt social behaviour with ToM task performance, in order to compare the characteristics of ‘Low’ and ‘High’ Compensators.

**Methods:**

A total of 136 autistic adolescents, from the ongoing Social Relationships Study, completed a range of cognitive tasks, the Autistic Diagnostic Observation Schedule (ADOS) and a self‐report anxiety questionnaire. Participants were assigned compensation group status; High Compensators demonstrated good ADOS scores despite poor ToM performance, while Low Compensators demonstrated similarly poor ToM, accompanied by poor ADOS scores.

**Results:**

High Compensators demonstrated better IQ and executive function (EF), but greater self‐reported anxiety, compared with Low Compensators. Such differences were not found when comparing individuals who had good versus poor ADOS scores, when ToM performance was good. Other core autistic characteristics (weak central coherence, nonsocial symptoms) did not differentiate the High and Low Compensators.

**Conclusions:**

IQ, EF and anxiety appear to be implicated in the processes by which certain autistic young people can compensate for their underlying ToM difficulties. This tendency to compensate does not appear to reflect the severity of ‘hit’ for ASD per se*,* suggesting that well‐compensated individuals are not experiencing a milder form of ASD. The construct of compensation in ASD has implications for research and clinical practice.

## Abstract 2

Read the Commentary on this article at doi: 10.1111/jcpp.12989

## Introduction

Autism Spectrum Disorder (ASD) is characterised by impairments in social communication and interaction and repetitive and restricted behaviours and interests (RRBIs; American Psychiatric Association, APA, 2013). It is heterogeneous in many ways, including developmental trajectory. For example, while most children with an ASD diagnosis continue to experience substantial social difficulties into adulthood, a subset may no longer fulfil diagnostic criteria (e.g. Fein et al., 2013; Gillberg, Helles, Billstedt, & Gillberg, 2016), thus appearing ‘socially adapted’, in overt behaviour at least.

It is currently unclear why some autistic individuals show better outcome (e.g. improved social skills) than others (for review, see Howlin & Magiati, 2017). Elsewhere we have explored the candidate mechanism of ‘compensation’ (Livingston & Happé, 2017). Our compensation framework suggests that when an individual's symptoms lessen, this may not necessarily signify alteration or alleviation of underlying cognitive deficits, but instead, an enhanced tendency to *compensate* for these deficits. Equally, when symptoms worsen, this may not signal decrement in the underpinning cognitive abilities, but instead, the breakdown of compensation, which was otherwise disguising or minimising cognitive difficulties. The outcome of compensation, therefore, is that an individual's behavioural presentation may appear less severe than otherwise predicted by their underlying cognitive abilities/atypicalities (Livingston & Happé, 2017).

The phenomenon of compensation may overlap partially with that of ‘camouflaging’ (Hull et al., 2017; Lai et al., 2017). Camouflaging refers to the behavioural modifications that autistic individuals may use to ‘blend in’ or appear neurotypical; for example, dressing like one's neighbour, suppressing repetitive behaviours or avoiding taxing social events. Compensation will also result in a more neurotypical behavioural presentation, however, it goes further than masking/suppression of autistic traits and, instead, involves alternative cognition to circumvent underlying cognitive difficulties. For example, while you might mask a difficulty in distinguishing lies from jokes by copying the behaviour of others (e.g. laughing), compensation would involve developing a conscious rule: when someone says a nonliteral statement and is laughing, it is likely a joke (else, it is likely a lie). In addition, while camouflaging tends to describe how the whole of one's autism is disguised, the compensation framework suggests that a single cognitive atypicality can be ‘compensated for’ (improving, e.g. social skills), leaving other atypical behaviours unchanged (e.g. RRBIs). Crucially, measuring compensation requires probing both behaviour and underlying cognition, in order to quantify the discrepancy between the two.

The construct of compensation in ASD has received almost no empirical attention. There has, however, been indirect discussion of compensation‐like phenomena; where *perceived* ability, as demonstrated in overt behaviour, is substantially better than *actual* ability, as measured on cognitive tasks. For instance, it has been widely proposed that difficulties in inferring others’ mental states, or ‘theory of mind’ (ToM), are responsible for the reduced social skills observed in ASD (e.g. Happé, 2015) and there is evidence to support this (e.g. Mazza et al., 2017; for overview, see Brunsdon & Happé, 2014). Compensation becomes relevant when considering that some autistic individuals demonstrate fairly good social skills, despite clear sociocognitive atypicalities, as measured by sensitive ToM tasks (e.g. anticipatory gaze based on false belief attribution; Senju, Southgate, White, & Frith, 2009; Schneider, Slaughter, Bayliss, & Dux, 2013). It is suggested that compensation allows individuals to use alternative cognitive routes, independent of ToM, to support good social skills in behaviour. These compensatory strategies, however, do not appear to allow for the effortless and flexible mentalising observed in neurotypical individuals. As such, this type of compensation is envisaged as shallow and fragile; it may not translate well across settings and is insufficient to pass sensitive ToM tasks (Livingston & Happé, 2017). It is possible that some autistic individuals might compensate more deeply, such that compensatory processes are sophisticated enough to support ToM task performance, although this has never been empirically tested (for more detail, see Livingston & Happé, 2017).

What are the individual differences that might contribute to compensation? We suspect that if compensation is to explain heterogeneity (e.g. in developmental trajectory, outcome), relevant cognitive abilities will be those that vary orthogonally with core autistic impairments; for example, IQ (Charman et al., 2011) and executive function (EF; Geurts, Sinzig, Booth, & Happé, 2014). Good IQ (e.g. Magiati, Tay, & Howlin, 2014) and EF (e.g. Pugliese et al., 2015) predict improvement in ASD symptoms across development, suggesting a role for these abilities in compensation. Johnson, Jones, and Gilga (2015) have outlined how higher‐order EFs might buffer against ASD‐related brain atypicalities, thus reducing the severity of autistic symptoms later seen in behaviour. Ullman and Pullman (2015) have also proposed that intact EF ability may facilitate retrieval of learned information or social ‘rules’ from memory in ASD.

Intriguingly, in the one study that has directly measured the discrepancy between social behaviour and underlying difficulties in ASD, termed ‘camouflaging’, individual differences in EF, but not IQ, contributed to camouflaging scores. Among autistic adults, Lai et al. (2017) measured camouflaging as the numerical difference between standardised scores of ‘external’ versus ‘internal’ measures; observer‐rated autistic symptoms (using Autism Diagnostic Observation Schedule, ADOS; Lord et al., 2000) versus sociocognitive ability (inferred from Reading the Eyes in the Mind task; Baron‐Cohen, Wheelwright, Hill, Raste, & Plumb, 2001) and self‐reported autistic traits. Camouflaging scores were positively correlated with inhibition‐based EF in females and depression in males.

This latter finding begs the question; is the outcome of compensation always positive? While “cushion[ing] the effect of the deficit” (Frith, 1991, p. 21) could facilitate many positive outcomes (e.g. independent living, employment), compensation might come at a cost. We speculate that compensating could be highly taxing, especially if it requires additional cognitive resources not otherwise used by neurotypical individuals for mentalising. Indeed, exhaustion associated with ‘appearing normal’ is alluded to in self‐reports from autistic individuals (e.g. Hull et al., 2017).

This study aimed to operationalise compensation as the discrepancy between sociocognitive ability (ToM performance) and observer‐rated social behaviour (ADOS), in a population‐derived sample of autistic individuals, thus overcoming issues of clinic bias (possible in Lai et al., 2017); the best compensators are less likely to come to clinical attention. We aimed to compare the cognitive (IQ, EF) and mental health (anxiety) characteristics of High and Low Compensation groups; the former reflecting good (low) ADOS scores despite poor ToM, and the latter, equally poor ToM alongside poor (high) ADOS scores. The two groups were also compared on other key cognitive (weak central coherence) and behavioural (nonsocial symptoms) features of ASD. To ascertain whether differences between High and Low Compensation groups reflect something specific about having poor versus good ADOS alongside poor ToM, individuals who had good ToM performance and either good or poor ADOS were similarly compared on the aforementioned characteristics.

Finally, we explored evidence for compensation within the autistic participants’ unaffected co‐twins. These individuals, despite often exhibiting some ASD‐related behaviours (Sucksmith, Roth, & Hoekstra, 2011) and neurocognitive atypicalities (e.g. Anzures, Goyet, Ganea, & Johnson, 2016), present neurotypically in behaviour. In light of the compensation framework, there remains an intriguing possibility that some co‐twins have compensated for an aetiological ‘hit’ for ASD such that they appear ‘unaffected’ at the behavioural level, despite underlying cognitive difficulties (see Livingston & Happé, 2017).

## Methods

### Participants

Participants were 136 adolescents (112 males) aged 10–15 years (*M *=* *13.28, *SD* = 0.93) who met diagnostic criteria for Autism Spectrum Disorder (ASD; *n *=* *101) or the Broader Autism Phenotype (BAP; *n *=* *35), alongside their unaffected co‐twins (*n *=* *67). Participants were part of the Social Relationships (SR) Study, a longitudinal investigation of twins with ASD originally identified from the population‐based Twins Early Development Study (Haworth, Davis, & Plomin, 2013; for SR Study sample ascertainment, see Colvert et al., 2015).

Autism Spectrum Disorder diagnoses were ascertained using the Autistic Diagnostic Interview‐Revised (ADI‐R; Lord, Rutter, & Le Couteur, 1994) and Autism Diagnostic Observation Schedule (ADOS; Lord et al., 2000). Autism Genetic Resource Exchange (http://research.agre.org/agrecatalog/algorithm.cfm) cut‐offs identified those with BAP. ASD and BAP groups were subsequently collapsed to form one ASD group (*N *=* *181) to reflect the broad spectrum specified in DSM‐5 (APA, 2013). Co‐twins were deemed unaffected if they failed to get a best estimate clinical diagnosis across multiple diagnostic sources (see Brunsdon et al., 2015).

Autism Spectrum Disorder participants were selected for this study on the basis of complete data for the ADOS and chosen ToM task. A total of 45 individuals were excluded due to missing data. This resulted in a final sample of 136 ASD participants, who did not significantly differ from the excluded group in terms of gender, χ^2^(1) = 0.26, *p *=* *.61, or SES, *t*(113) = 0.99, *p *=* *.32. The final sample included significantly fewer individuals with IQ < 70, χ^2^(1) = 23.58, *p *<* *.001 (φ = 0.36, *p *<* *.001), likely due to the verbal demands of the ToM task.

### Measures

#### Social‐economic status

Social‐economic status (SES) was a composite score from the Twins Early Development Study database (see Hanscombe et al., 2012).

#### Intellectual ability

Full‐scale IQ (FSIQ) was measured using the four‐subtest version of the Wechsler Abbreviated Scale of Intelligence (WASI; Wechsler, 1999). Verbal (VIQ) and nonverbal IQ (NVIQ) were calculated from WASI subtests. Two participants could not complete the WASI and instead, FSIQ was estimated from the British Picture Vocabulary Scales‐II (Dunn, Dunn, Whetton, & Burley, 1997) and Raven's Coloured Progressive Matrices (Raven, Raven, & Court, 1998), with the former reflecting an estimate of VIQ and the latter, NVIQ.

#### Autistic symptoms

Autistic symptoms were measured using Module 3 of the observer‐rated Autistic Diagnostic Observation Schedule (ADOS; Lord et al., 2000). For the present analyses, ADOS scores reflecting social symptoms only (henceforth, ‘Social ADOS’) were used as nonsocial symptoms are putatively underpinned by cognitive atypicalities distinct from ToM (Brunsdon et al., 2015). ADOS scores reflecting nonsocial symptoms only (henceforth, ‘Nonsocial ADOS’) were used in some analyses.

#### Theory of mind

Theory of mind (ToM) was measured using the computerised Frith‐Happé Animations (Abell, Happé, & Frith, 2000), where participants give verbal descriptions of four sequences of a triangle attempting to influence the mental state of another triangle. These descriptions were rated on a 3‐point accuracy scale (0 = completely inaccurate, 1 = misses the critical point and 2 = key mental state correctly recognised), such that scores ranged from 0 (poor ToM) to 8 (good ToM). Interrater reliability between the original rater and three additional raters (calculated for 15% of the total SR Study sample) ranged from 91% to 95%.

#### Other cognitive measures

Executive function (EF) was measured using a battery of tasks, covering inhibition (Luria Hand Game; Hughes, 1996), set shifting (Intradimensional/Extradimensional Task; Hughes, Russell, & Robbins, 1994) and planning (Planning Drawing Task, PDT; Booth, Charlton, Hughes, & Happé, 2003). On the assumption that EF is a multifaceted cognitive ability and with no a priori hypotheses about which aspects of EF might be most relevant to compensation, a composite EF score was created by calculating performance for each EF task as a % of maximum possible score and averaging performance across the three tasks. Detail focus, or (weak) central coherence (CC), was measured using the Sentence Completion Task (Booth & Happé, 2010) and the PDT (Booth et al., 2003).

#### Anxiety

Self‐ and parent‐reported anxiety was measured using the Anxiety section of the 47‐item Revised Child Anxiety and Depression Scale (Chorpita, Yim, Moffitt, Umemoto, & Francis, 2000), including five subscales: social phobia, panic disorder, generalised anxiety disorder, separation anxiety disorder and obsessive‐compulsive disorder. Total anxiety scores reflected the sum of all subscales. These data have previously demonstrated medium‐to‐strong internal consistency (Hallett et al., 2013). Total and subscale scores were calculated as a proportion of the maximum possible score based on items answered, provided < 25% of data were missing for that subscale.

### Procedure

See Brunsdon et al. (2015) for details of procedure for cognitive tasks and diagnostic assessments at home visits. Anxiety questionnaires were given to participants and their parents to post back.

### Statistical analyses

**Figure 1:**
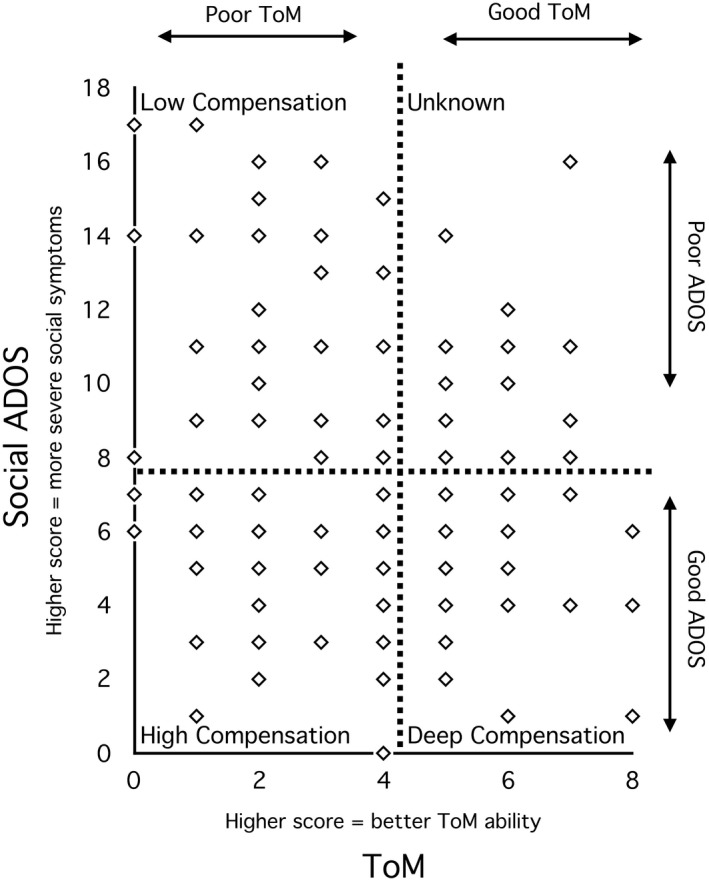
The relationship between ToM and social symptoms from the Autistic Diagnostic Observation Schedule (Social ADOS) in Autism Spectrum Disorder (ASD) participants. Dashed lines represent splits at (a) median ToM score of typically developing participants, (b) median Social ADOS score of ASD participants, resulting in four quadrants; Low Compensation, poor ToM+poor ADOS; High Compensation, poor ToM+good ADOS; Deep Compensation, good ToM+good ADOS (compensation is deep enough to support ToM task performance); Unknown, good ToM+poor ADOS (an unusual profile)

Participants (*N *=* *136) were first split based on median ToM performance (*Mdn* = 4) of 75 age‐, IQ‐ and SES‐matched typically developing (TD) participants from the SR Study, creating groups with good ToM (> TD median) and poor ToM (≤ TD median). Participants were also split by median Social ADOS score of the ASD participants (*Mdn* = 7), forming groups with good ADOS (≤ ASD median) and poor ADOS (> ASD median) scores. The four resulting groups reflect the quadrants of the ToM by ADOS dimensional space (Figure 1).

Given this study's primary interest in comparing Low and High Compensators, planned *t*‐test comparisons between these groups were conducted for the following variables using the regress function on STATA: FSIQ, VIQ, NVIQ, EF, anxiety (self‐ and parent‐report), CC and Nonsocial ADOS. Equivalent comparisons were performed between the two groups demonstrating good ToM (Deep Compensation and Unknown) to ascertain whether any observed differences between Low and High Compensation groups were simply a general function of ADOS scores. Where variables were correlated with VIQ (EF, anxiety and CC), comparisons were adjusted for VIQ.

Some variables (EF, CC, Nonsocial ADOS and self‐reported anxiety) demonstrated a nonnormal distribution and were transformed using the Van der Waeden technique. In some instances, data were still nonnormal and nonparametric equivalent comparisons were performed instead. Statistical analyses were performed using STATA 14 and were adjusted for clustering within twin pairs using robust standard errors (Williams, 2000).

## Results

Key characteristics of the four groups (Low Compensation, High Compensation, Deep Compensation and Unknown) are shown in Table 1. The groups did not significantly differ in terms of age, SES and the distribution of gender (males/females), twin zygosity (monozygotic/dizygotic) or co‐twin status (ASD/unaffected). Low and High Compensation groups did not significantly differ in terms of poor ToM (*p *=* *.09, *d *=* *0.4) and Deep Compensation and Unknown groups had equivalently good ToM (*p *=* *.53, *d *=* *0.18), fulfilling a critical assumption on which the study design lies.

**Table 1:** Participant characteristics

	Low Compensation		High Compensation		Deep Compensation		Unknown		ANOVA		
	*N*	*M (SD)*	*N*	*M (SD)*	*N*	*M (SD)*	*N*	*M (SD)*	*F*	*p*	*η*²
Age (years)	40	13.29 (0.84)	41	13.24 (0.99)	33	13.21 (0.96)	16	13.46 (0.95)	0.33	.81	0.01
SES (composite score)	27	−.08 (0.76)	31	0.20 (0.69)	23	0.15 (0.79)	14	0 (0.76)	0.88	.46	0.04
Social ADOS	40	12.13 (2.99)	42	4.43 (1.94)	34	4.85 (1.65)	20	10.35 (2.06)	99.54	<.001	0.75
ToM	40	2.15 (1.17)	42	2.67 (1.39)	34	5.91 (0.93)	20	5.75 (0.85)	137.53	<.001	0.80
Current ADI‐R^a^	39	16.28 (7.99)	40	11.03 (6.23)	34	12.15 (7.18)	20	12.25 (8.00)	3.41	.02	0.09
Male:Female^b^		4.71:1		3.67:1		4.67:1		9:1	–		
DZ:MZ twin^b^		2.08:1		2:1		4.5:1		1.71:1	–		
Co‐twin has ASD:Unaffected^b^		1.92:1		0.95:1		1.14:1		1.5:1	–		

ANOVAs are adjusted for clustering within twin pairs.

Low Compensation, poor ToM+poor ADOS; High Compensation, poor ToM+good ADOS; Deep Compensation, good ToM+good ADOS; Unknown, good ToM+poor ADOS; DZ, dizygotic; MZ, monozygotic; Unaffected, does not meet ASD or BAP criteria.

Higher scores reflect higher SES/more severe ADOS or ADI‐R symptoms/better ToM ability.

a ADI‐R scores reflect current parent‐reported social symptoms (sum of domains A and B from Current Behaviour Algorithm of the Autism Diagnostic Interview‐Revised; Lord et al., 1994).

b Chi‐Square tests, all group interactions nonsignificant (*p *>* *.40).

Results of the planned *t*‐test comparisons are shown in Table 2. Effect sizes are reported in terms of Cohen's *d* (0.2 = small, 0.5 = medium and 0.8 = large; Cohen, 1988). High Compensators scored significantly higher than Low Compensators in terms of FSIQ, VIQ (but not NVIQ), EF and self‐ (but not parent‐) reported anxiety. These groups did not differ significantly in terms of CC or Nonsocial ADOS. Deep Compensation and Unknown groups demonstrated no significant differences in any of the above variables.

**Table 2:** Planned comparisons between Low and High Compensation groups and between Deep and Unknown groups

	Low Compensation		High Compensation					Deep Compensation		Unknown			
	*N*	*M (SD)*	*N*	*M (SD)*	*p*	*d*	Direction of effect	*N*	*M (SD)*	*N*	*M (SD)*	*p*	*d*
Full‐scale IQ	40	85.54 (20.60)	42	94.60 (17.58)	**.032**	**0.47**	**High > Low**	34	101.88 (14.75)	19	97.11 (16.08)	.24	0.31
VIQ	40	84.09 (18.44)	42	92.71 (16.64)	**.028**	**0.49**	**High > Low**	34	97.94 (17.48)	19	95.11 (18.57)	.56	0.16
NVIQ	40	89.23 (22.86)	42	97.50 (18.80)	.071	0.40	–	34	106.21 (13.83)	19	99.58 (13.12)	.07	0.49
EF^a^_max = 100_	33	74.81 (13.91)	38	83.49 (12.47)	**.022**	**0.65**	**High > Low**	31	82.19 (11.09)	17	80.75 (12.43)	.86	0.07
Total Anxiety self‐reported^a^_max = 108_	26	23.29 (16.48)	29	31.29 (15.38)	**.054**	**0.53**	**High > Low**	25	26.66 (14.65)	15	23.21 (16.57)	.17	0.22
SP^a^_max = 27_	26	8.38 (5.61)	30	11.58 (5.71)	**.032**	**0.58**	**High > Low**	25	9.80 (5.24)	15	9.45 (5.92)	.53	0.07
PD^b^_max = 27_	26	3.29 (3.44)	30	5.45 (3.78)	**.015**	–	**High > Low**	25	4.37 (3.80)	15	3.04 (4.64)	.10	–
GAD _max = 15_	26	3.62 (3.58)	30	5.13 (2.66)	**.027**	**0.65**	**High > Low**	25	4.61 (3.21)	15	3.73 (2.87)	.14	0.28
SAD^b^_max = 21_	26	3.42 (3.35)	29	4.32 (3.54)	.26	–	–	25	3.36 (2.38)	15	3.04 (3.60)	.41	–
OCD^b^_max = 18_	26	4.58 (4.44)	30	4.54 (2.66)	.43	–	–	25	4.52 (3.33)	15	3.95 (3.80)	.55	–
Total Anxiety parent‐reported^a^_max = 108_	30	24.15 (12.22)	29	26.73 (13.53)	.42	0.20	–	27	23.30 (12.49)	14	22.20 (12.01)	.38	0.09
CC SCT^a^_max = 20_	39	9.85 (3.11)	41	11.07 (3.00)	.13	0.44	–	34	10.71 (2.41)	20	10.15 (3.03)	.70	0.17
CC PDT^a^_max = 12_	40	1.78 (0.92)	42	1.81 (0.99)	.86	0.04	–	34	1.97 (0.90)	20	1.95 (0.83)	.83	0.02
Nonsocial ADOS^b^_max = 8_	40	2.80 (2.65)	42	1.83 (1.62)	.20	–	–	34	2.41 (2.20)	20	2.10 (2.02)	.67	–

VIQ, verbal IQ; NVIQ, nonverbal IQ; EF, executive function; SP, social phobia; PD, panic disorder; GAD, generalised anxiety disorder; SAD, separation anxiety disorder; OCD, obsessive‐compulsive disorder; CC, central coherence; SCT, sentence completion task; PDT, planning drawing task; Low Compensation, poor ToM+poor ADOS; High Compensation, poor ToM+good ADOS; Deep Compensation, good ToM+good ADOS; Unknown, good ToM+poor ADOS.

Descriptive data are raw scores. Planned comparisons are based on transformed scores and adjusted for clustering within twin pairs. Bold values represent the comparisons that reached, or almost reached, statistical significance.

Higher scores reflect better EF/greater self‐ or parent‐reported anxiety/greater CC (i.e. less detail focus)/more severe nonsocial symptoms.

a All comparisons for EF, anxiety (self‐ and parent‐report), CC SCT and CC PDT were adjusted for VIQ.

b Mann–Whitney *U* comparisons performed due to nonnormal distribution of data after transformation (*d* values not reported).

The Unaffected Co‐twins group (*n *=* *67) was investigated in an exploratory manner. The Unaffected Co‐twins spanned almost the full range of ToM scores (*Mdn* = 5, *range* = 1–8) and the lower half of possible ADOS scores (*Mdn* = 0, *range* = 0–9). A total of 29/67 had ToM scores the same as or below the TD median (*Mdn* = 4), such that they lay in the quadrant space for the High Compensation ASD group, with 9 of these demonstrating ToM as poor as the lowest scoring quarter of the ASD participants (ToM score = 0–2).

## Discussion

This study aimed to explore an intriguing subgroup of young people with ASD, who, despite continued difficulty understanding other minds, display relatively few social symptoms in behaviour, and are therefore putatively ‘High’ Compensators. Our results suggest that, compared to age‐ and SES‐matched autistic participants with equally poor ToM but substantially more severe social symptoms (‘Low’ Compensators), High Compensators demonstrated higher verbal (but not nonverbal) IQ, better EF and greater self‐reported anxiety. Crucially, the differences between High and Low Compensators were not simply a function of levels of ADOS‐assessed social skills; the other two groups in this sample (Deep Compensation, Unknown), similarly split on ADOS but demonstrating good ToM, were comparable in terms of the aforementioned variables. Therefore, it appears to be the specific combination of performing well on the ADOS, despite poor ToM, that is associated with higher IQ, EF and anxiety.

Although the present associations cannot speak to causal effects, it seems plausible that individual differences in IQ, particularly verbal IQ, could influence compensation, for example, via the use of learned social rules and scripts. Whether the good ADOS performance of the High Compensators was reflecting surface‐level ‘masking’, as suggested by the camouflaging literature (e.g. suppressing autistic behaviours) or instead, active strategies that allow one to appear socially skilled (i.e. compensation; e.g. deliberate eye contact) warrants further investigation. Indeed, there may be some items on the ADOS that could be passed through simply suppressing certain behaviours and others that would require an active compensatory strategy.

The EF results are in line with the suggestion that EF ability has an important compensatory function (Johnson, 2012; Ullman & Pullman, 2015). In particular, our data suggest that there is something particularly beneficial about having good EF/IQ when underlying ToM ability is poor and that EF might have an influence over and above (verbal) IQ, in line with Lai et al. (2017). However, there remain important questions about whether these cognitive abilities necessarily precede compensation. It is plausible that an early form of compensation might facilitate social interaction, thereby providing an improved learning environment for acquiring the skills measured in IQ/EF tests.

The results also support the possibility of a cost to compensation. The fact that heightened (self‐reported) anxiety was found in the High versus Low Compensators, while controlling for verbal IQ, suggests that there was not simply a bias towards individuals with higher IQ having greater ability to self‐report. This link between compensation and anxiety, however, could be directional or bidirectional. On the one hand, compensating for core ToM difficulties could have downstream effects on well being. Compensation may require high amounts of additional cognitive resources (e.g. EF) and therefore be prone to break down when these resources are depleted, hence promoting fatigue and anxiety. Alternatively, those individuals who experience greater anxiety might be more motivated to compensate, or anxiety could mask autistic symptoms in the ADOS. Finally, it is intriguing that group differences between High and Low Compensators were not found for parent‐reported anxiety. On inspection of the mean values, it appears that lack of significant differences may be due to parents underestimating anxiety among High Compensators.

An alternative explanation for the present findings would be that High Compensators are simply experiencing a milder form of ASD, i.e. that they have a lesser cognitive or aetiological ‘dose’ of ASD for which to compensate. This, however, seems unlikely for a number of reasons. High Compensators exhibited a ToM deficit considerably more severe than that of many other participants. In addition, there is no doubt across multiple diagnostic measures (see *Methods*) that High Compensators did have ASD, despite doing well on the ADOS. Finally, the data suggest that High and Low Compensators were equivalent in the manifestation of other core autistic features, such as nonsocial symptoms and detail focus (or weak central coherence). Interestingly, the groups were as likely to have a co‐twin who also had ASD, suggesting similar genetic ‘hit’ for ASD. Therefore, instead of High Compensators having a ‘milder’ form of ASD, they appear to be experiencing similar underlying cognitive difficulties (i.e. ToM difficulties) to Low Compensators but have an enhanced ability/propensity to compensate in their overt behaviour, at least in the context of the ADOS.

Results from the Unaffected Co‐twins group suggest that a substantial proportion had underlying ToM difficulties, despite appearing ‘neurotypical’ in behaviour, highlighting the intriguing possibility that genetically predisposed (but unaffected) individuals may be compensating for some residual cognitive difficulties. As these individuals did not meet diagnostic criteria for ASD, any compensation taking place is probably reliant on fairly sophisticated strategies (deep compensation), rather than learned, inflexible social rules (shallow compensation).

A major strength of this study is that, due to the population‐based nature of the SR Study, not all autistic participants had a prior clinical diagnosis, allowing for compensation to be explored among those that had not come to clinical attention. There are, however, a few limitations. Although the overall sample size was reasonable, segregating participants into four groups substantially reduced statistical power. It could be argued that finding no group differences between Deep Compensation and Unknown groups was due to these groups having smaller sample sizes, however, the effect sizes comparing the two were also small. Equally, although High and Low Compensators did not differ statistically in their ToM ability, we acknowledge the possibility that our group sizes may have been too small to detect a real difference. In addition, although our predictions concerned Low versus High Compensators, not taking into account multiple comparisons across the four groups may have increased Type 1 error. Finally, given the verbal nature of the ToM task, the majority of autistic participants with low IQ from the SR Study could not be included, making our findings unrepresentative of this portion of the autism spectrum.

The current findings have important clinical and theoretical implications. First, they suggest that the ADOS, a one‐to‐one assessment in a quiet setting with a trained individual, may not always reveal real underlying sociocognitive difficulties for some autistic individuals. It remains to be seen whether compensatory strategies used in the ADOS translate to unstructured everyday social situations, such as school or work environments (Dean, Harwood, & Kasari, 2017). It is possible that for certain individuals, any compensation is fairly shallow and may break down in complex situations where social cues are unpredictable, ambiguous and/or fast‐paced (see Livingston & Happé, 2017). With this said, in the current sample, the High Compensators also appeared to have better observable social skills than Low Compensators according to parent ratings (Current ADI‐R; see Table 1), which should capture the individual's behavioural profile across multiple settings.

The findings also stress the importance of cognitive, alongside behavioural assessments, when understanding individual differences in ASD. In this study, participants’ ToM performance added substantial information, in combination with ADOS scores, in predicting key cognitive and mental health characteristics. The notion of compensation might shed light on underdiagnosis of ASD, for example, among females. This study did not find females were more likely to be High versus Low Compensators, although power was limited (24 females). The findings also highlight that self‐report may be particularly informative, at least for detecting anxiety among the subgroup of young people with ASD who tend to compensate. This anxiety may be overlooked by parents. Finally, the findings also inspire theoretical questions about (a) whether interventions aimed at improving social skills are actually tapping compensatory mechanisms, and (b) whether such interventions necessarily have a positive outcome, for example, for mental health.

On a final note, as previously outlined, a sensitive cognitive probe is essential for being able to measure compensation, defined as the difference between behavioural presentation and underlying cognitive ability (Livingston & Happé, 2017). The ToM task used in this study is sufficiently novel to be relatively immune to learned strategies, and is among the most sensitive in the field for revealing underlying ToM difficulties among autistic individuals. However, as some participants (e.g. those in the Deep Compensation group) performed reasonably well on the ToM task, the possibility remains that participants’ task performance could itself be supported by some compensatory processes. Future research aimed at refining the sensitivity of ToM measures, as well as exploring the distinction between deep and shallow compensation, is required; for example, establishing whether ‘deep compensators’ do well across a range of (implicit and explicit) ToM tasks and whether difficulties are revealed with increasing task sensitivity.

## Conclusions

This study operationalised compensation as improved behavioural presentation of ASD, despite persistent core cognitive deficits. We used observer‐rated social symptoms and ToM task performance as measures of the behavioural and cognitive levels, respectively, although ToM deficit is just one ASD‐related cognitive atypicality (of many) that may be compensated for and could be studied. The results suggest that IQ, EF and self‐reported anxiety are implicated in the processes of compensating for ToM difficulties in ASD. Further research, including a developmental longitudinal approach incorporating multiple ToM tasks, should aim to delineate the exact nature of compensatory mechanisms, so that the theoretical and clinical relevance of the concept of compensation to understanding ASD can be established.

**Key points**

- We operationalised a novel definition of ‘compensation’ in ASD as improved behavioural presentation (here, good social skills), despite persistence in core cognitive deficits (here, theory of mind difficulties).
- A subgroup of young people with ASD compensate well, displaying reasonable social skills in behaviour, despite clear difficulties on a theory of mind task.
- Greater ability/propensity to compensate is associated with higher verbal IQ, better executive function and greater self‐reported anxiety.
- These results have implications for understanding underdiagnosis and mental health difficulties in ASD, as well as the mechanisms that may underpin social skills training.
